# Modeling the formation of Sedan Crater using the FLAG and HOSS codes

**DOI:** 10.1038/s41598-025-06621-6

**Published:** 2025-07-01

**Authors:** Sebastian F. Henderson, Angel Padilla, Wendy K. Caldwell, Esteban Rougier, Zhou Lei, Matthew R. Sweeney, Philip H. Stauffer

**Affiliations:** 1 X Computational Physics Division, Los Alamos National Laboratory, Los Alamos, NM 87545 USA; 2 Earth & Environmental Sciences Division, Los Alamos National Laboratory, Los Alamos, NM 87544 USA

**Keywords:** Mathematics and computing, Geophysics

## Abstract

Numerical modeling of explosion crater formation requires accounting for complex physical processes. Numerical validation of explosion cratering is an important step in modeling and requires experimental data for comparison. Models using discrete elements and continuum models have both benefits and drawbacks to their approaches. In this work, we consider both an arbitrary Lagrangian–Eulerian (ALE) hydrocode and a finite discrete element method (FDEM) approach to modeling the formation of the Sedan crater, the largest human-made crater in the United States. The Sedan crater formed from an underground nuclear detonation in the Nevada desert as part of Project Plowshare. Our models show that the continuum approach of the hydrocode matched well compared to early test time prior to the mound rupture and subsequent fireball venting, when most of the alluvium exhibited fluid behavior. Our FDEM approach matched the final crater dimensions well, after material had settled back into the crater, when material strength and solid mechanics play key roles. Our work shows how leveraging the benefits of multiple numerical approaches can lead to better understanding of complex physical problems, especially problems with limited experimental data. By using a continuum approach to early-time hydrodynamics and an FDEM approach to later-time solid mechanics, we can better understand the different physical regimes of explosion crater formation.

## Introduction

Explosion cratering involves complicated physical processes, including shock wave propagation, material stress and strain responses, crater excavation and modification, and material failure. Explosion craters can occur naturally, often from volcanic activity^1^, or can be human-made, from nuclear or chemical explosive detonations^2–4^. Because of the practical risks associated with studying the formation of these craters, as well as the dearth of new experimental data, accurate numerical modeling is essential for continued study of explosion crater formation. More accurate modeling of explosion crater formation can expand our knowledge of shock wave propagation in rocky materials and can aid our abilities to perform predictive modeling of volcanic explosions and underground detonations. However, explosion cratering is a difficult problem to model numerically because of the range of coupled physical and chemical processes and compressible flow dynamics. To ensure the accuracy of numerical codes, these codes must be validated against natural and experimental analogs. Historical nuclear and chemical explosion tests are often targeted for code validation efforts, which are separate thrusts from other studies of crater formation that focus on empirical and scaling relationships^5,6^. The purpose of this paper is to present validation of two state-of-the-art three-dimensional multiphysics codes against results from an underground nuclear explosion that resulted in one of the largest craters ever made by humans and to discuss the merits and challenges associated with different modeling strategies used to capture crater-forming explosions.

After performing more than 1000 nuclear tests starting with Trinity test in 1945, the United States stopped testing nuclear explosions in September of 1992^7^. Hence, computational models, with validation by comparison to historic data from nuclear testing, are essential for understanding underground nuclear explosions. The Sedan test provides validation data for modeling underground nuclear explosions and the resulting craters at spatial scales not attainable in laboratory environments or through the use of conventional explosives. One stated purpose of the Sedan test was the development of scaling laws so that the data could be extrapolated into regimes with much higher energy related to meteor impacts^8^. Laboratory-scale experiments and conventional explosions are orders of magnitude smaller than the desired high-energy range and do not scale linearly^9^.

Sedan Crater is the largest human-made crater in the United States and the second largest human-made crater in the world, with an apparent depth of about 98.5 m, a true depth of 245 m, and an apparent diameter of about 372 m^7,8^. The true depth includes the final crater depth as well as the excavated material, similar to what would be considered the transient crater for an impact crater. The Sedan explosion was part of Operation Storax, but was also part of Project Plowshare, a project designed to study peaceful applications of nuclear explosion technology, including excavating large craters^10^. Explosives had previously been used for such applications, and Plowshare sought to answer similar questions related to nuclear explosions rather than chemical explosions^10^. In the Sedan test, a nuclear device was detonated at a buried depth of approximately 194 m. The test was located in the deep alluvium of Yucca Flat at the Nevada National Security Site, formerly known as the Nevada Test Site. The resulting explosion had the energy equivalent of 104 kilotons (kt) of TNT^7,8^. Sedan was the largest cratering test in Project Plowshare^4,7^.

To simulate crater formation associated with the Sedan test, we consider two numerical approaches: a continuum hydrodynamics code (hydrocode) approach (FLAG) and a combined finite-discrete-element approach (HOSS). These two approaches, with both underlying numerical codes developed and maintained by Los Alamos National Laboratory (LANL), have previously been shown to be effective in modeling the formation of craters from high-velocity impacts^11–14^. Both of these approaches performed well when compared to other simulation tools used to model impact crater formation when applied to simulate the same verification problems^11,12,15^. We note that in the original Pierazzo study from 2008, relative errors of around 30% were reported by hydrocodes commonly used to model cratering^15^.

Given the publicly available data from the Sedan test, our metrics of success include mound venting time, true and apparent crater depth, and final crater diameter. The true, often referred to as the transient, crater depth is listed in the report as 245, and the apparent, or final, depth is listed as 98.5 m^7,8^. Thus, successful depth predictions will lie between these values. Using both the transient and final crater depths allows us to validate crater depth throughout all simulation time rather than just relying on the final crater depth and is an approach used in other cratering simulation studies^16–18^. Because the report does not list a true/transient crater diameter, we use the final crater diameter as a lower bound. Successful diameter predictions will be at least as large as the final crater diameter, with early simulation times expected to be as much at 2.5 times the final diameter size, about 925 m, using the transient-to-final depth ratio as an upper bound for the transient diameter.

By using two numerical approaches to these models, we aim to determine the validity of each method separately and gain insight into a multi-code approach that leverages the strengths of each approach. We anticipate that the hydrocode FLAG will provide to have higher fidelity in matching transient crater dimensions and modeling early simulation times, when all material exhibits fluid behavior, while the FDEM code HOSS will result in better matches to final crater dimensions and later simulation time, when material strength dominates the crater modification stage. A detailed description of the FLAG and HOSS codes appears in “Methods”. These two numerical methods are used for a variety of applications and physics regimes, and cratering simulations provide an overlap in their capabilities.

Here, we present simulations by both FLAG and HOSS for the Sedan crater formation, from detonation through crater modification. In “Methods”, we describe the underlying numerics of these methods, their features, and their prior usage. We also describe the materials we used for this study and the computational domain and initial conditions of our simulations. In “2D axisymmetric results”, we present 2D axisymmetric results of FLAG simulations. We include 2D results to assist in quantifying uncertainties between 2D and 3D simulations. In “3D results”, we present 3D results from both FLAG and HOSS. In “Discussion”, we discuss our results in the broader context on explosion cratering. Finally, in “Conclusions and future work”, we discuss conclusions and possible future work.

## Methods

We use the LANL-developed hydrodynamics code (hydrocode) FLAG^19–21^ (Free LAGrange) and the separate LANL-developed hybrid multiphysics software package HOSS (Hybrid Optimization Software Suite)^22^ to simulate the Sedan event.

FLAG is a finite-volume arbitrary Lagrangrian–Eulerian (ALE) hydrocode with multi-physics modeling capabilities in 1–3 spatial dimensions. FLAG has the ability to model both fluids and solids and can incorporate a variety of equations of state (EOS) and material models^23^.

The FLAG hydrocode solves the Euler equations:

$$\begin{aligned} \dfrac{\rho D {\textbf {u}}}{Dt}&= - \nabla P \\ \dfrac{D\rho }{Dt} + \rho \nabla \cdot {\textbf {u}}&= 0 \\ \dfrac{dE}{dt} + P\dfrac{dv}{dt}&= 0 \end{aligned}$$

with a second-order finite-volume scheme to adhere to conservation laws, where $\rho$ is density, ${\textbf {u}}$ is velocity, *P* is pressure, *E* is energy per unit mass, *V* is volume, and $\dfrac{D}{Dt}$ is the Lagrangian differential $\left( \dfrac{\partial }{\partial t} + {\textbf {u}} \cdot \nabla \right)$^24^. FLAG supports fully unstructured grids and is massively parallel^23^.

FLAG is used in a diverse range of physics problems, varying in temporal and spatial scales by several orders of magnitude^16,25–28^. In particular, FLAG has been shown to be accurate modeling impact craters through multiple validation^11,12,17,18,29^ and verification studies^12,18,30^. These previous studies have shown how FLAG is able to match crater depth, diameter, and aspect ratio at relative errors smaller than those from other common cratering codes in earlier benchmarking studies. Additionally, this previous work shows shock pressure decay in 1, 2, and 3 spatial dimensions in both strengthless FLAG simulations and simulations testing a variety of material models^12,18^ and porosity levels using the P-$\alpha$ model^31^ to evolve porosity^16–18,25,26,32^. FLAG has also been shown to preserve scaling relationships and hydrodynamic similarity in cratering simulations^30^.

HOSS is a LANL-developed multi-physics simulation platform that is based on the combined finite-discrete element method (FDEM)^33^ and integrates a computational fluid dynamics (CFD) solver^34^. HOSS is fully parallel^35^, and it can handle models in 1D spherical symmetry, 2D Cartesian, 2D axi-symmetry (*r*–*z*) , 2.5D (i.e., shells) , and 3D^22^. As its name indicates, FDEM merges the advances achieved within the Finite Element Methods (FEM) and the Discrete Element Method (DEM)^33,36–38^. As a result, a key advantage of HOSS is that it uses finite displacements, finite rotations, and finite strain-based deformability combined with a wide range of constitutive material laws^39–41^. All these components work in conjunction with discrete-element-based transient dynamics, contact detection, contact interaction solutions, and objective discrete crack initiation and crack propagation solutions^42–44^. HOSS has been applied to a diverse set of problems spanning from millimeter- to kilometer-length scales and micro-seconds to minutes in the time scale^45–52^.

The governing equation in discretized form being solved within the FDEM framework is

$$\begin{aligned} {\textbf {M}}\dfrac{d^2 {\textbf {x}}}{dt^2} + {\textbf {C}}\dfrac{d {\textbf {x}}}{dt}&= {\textbf {f}} \end{aligned}$$

where ${\textbf {x}}$ and ${\textbf {f}}$ are the displacement and the equivalent nodal force load vectors, respectively, while ${\textbf {M}}$ and ${\textbf {C}}$ are the mass and damping matrices^33^. FDEM resolves the equation above using an explicit time integration scheme; in the case of HOSS, the central difference time integration scheme is employed^53^.

### Simulation setup

Given the large amount of energy of the explosion, we modeled the Sedan event as a Taylor–von Neumann–Sedov blast wave problem^54^. At the depth of burial of the Sedan nuclear device, a spherical cavity filled with high energy, high density gas replaces the existing alluvium. The simulation carries out the time evolution of the gas as it expands radially outward, pushing the surrounding alluvium media to create the Sedan crater. In each simulation, the source was modeled using the pill source approach^55^, in which a vaporization radius of $r_v=2.0W^{(1/3)}$^56^, with *W* being the yield of the nuclear event in kilotons, was adopted. For Sedan, with a yield of $W=104$ kt^8^, we obtain $r_v=9.4$ m. Using Holsapple’s crater formation time formula from^57^, we expect the Sedan crater to take between 3.3 and 4.2 seconds to form.

### FLAG setup

**Figure 1:**
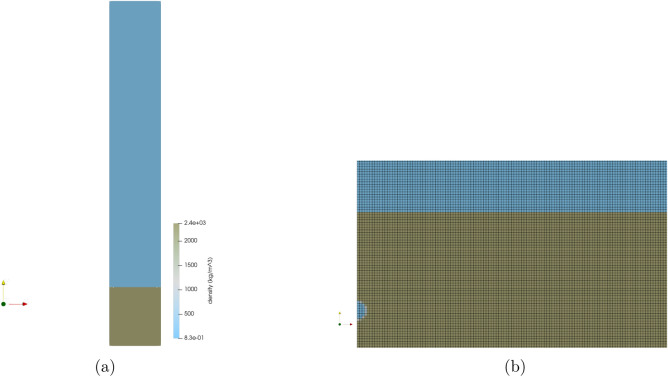
FLAG 2D axisymmetric simulation of the Sedan event at initialization. Figure created with ParaView 5.10.1 (https://www.paraview.org/).

The Sedan event was modeled in FLAG in both 2D axisymmetric and 3D geometries. The 2D simulation ran with a zone size of 5 m with 600,000 zones and a source composed of 28 zones. The computational domain was 1.5 km wide and 10 km high. The additional height of the computational domain was included to avoid boundary effects from the excavated crater material hitting the top boundary and falling back into the crater. Figure 1 shows the 2D FLAG simulation at initialization. Figure 1a shows the entire computational domain, colored by density. The alluvium is shown in brown at the bottom of the domain, and the surrounding air appears in blue. Figure 1b shows a zoomed-in view of the computational mesh at initialization. The source is shown as a blue circle in the brown alluvium. The surrounding air appears at the top in blue.

The 3D FLAG simulation ran with varying zone sizes from 2 m to 100 m with 8.1992 million zones total with a source composed of 656 zones. The computational domain spanned 3 km in all three spatial dimensions. The hydro boundary conditions in both setups allow for the motion of points along the respective boundary. The initial conditions and parameter values used as input for the start of the FLAG simulations are shown in Table 1. A constant gravity of $g = -9.81$ m/s$^2$ was assumed in the *y* and *z* directions, respectively, for 2D and 3D simulations. To model the behavior of the alluvium, the tabular SESAME EOS for Nevada alluvium (dirt) was chosen, using pressure $p_{\text {dirt}} = 1{\text {e}}5$ Pa and density $\rho _{{\text {dirt}}} = 2350$ kg/m$^3$ to initialize^58^, augmented with a bi-linear pressure ramp, with a first-segment bulk modulus of $K = 1.14142{\text {e-1}}$ MPa^59^. The strength model chosen was an isotropic elastic-plastic model. A constant gamma gas EOS, with $\gamma = 1.4$, was chosen to model the air, using pressure $p_{\text {air}}= 1e5$ Pa and density $\rho _{\text {air}} = 1.29$ kg/m$^3$ to initialize. The same gamma gas EOS was chosen to model the source, using density $\rho _{\text {source}}= 2350$ kg/m$^3$^58^ and energy $E = 8.1\text {e}6$ J^8^ to initialize. Because we are using a tabular EOS rather than an analytic EOS, we made a slight modification to the initial density to ensure we could initialize at the correct temperature and pressure. SESAME EOS tables are derived from experimental data, and thus it is not uncommon to need to make initialization modifications as the EOS are optimized for the high-temperature, high-pressure regimes attained through shock experiments^58^. A von Neumann–Richtmyer artificial viscosity was selected to remove numerical shock discontinuities across zones^60^.

**Table 1:** FLAG parameters and initial conditions.

Material property	FLAG initial value
Alluvium (solid)	
EOS	SESAME ID 7112^58^
Pressure	1e5 Pa
Density	2350 kg/m$^3$^58^
Bulk modulus	1.14142e-1 MPa^59^
Yield stress	3e5 Pa^61,62^
Shear modulus	7.3e8 Pa^59^
Melt temperature	1356 K
Tensile failure	1.2e5 Pa^61,62^
Air (gas)	
EOS	$\gamma$-law gas
Pressure	1e5 Pa
Density	1.2922 kg/m$^3$
$\gamma$	1.4
Temperature	273 K
Source (gas)	
EOS	$\gamma$-law gas
Density	2350 kg/m$^3$^58^
$\gamma$	1.4
Energy	8.1e6 J^8^

**Figure 2:**
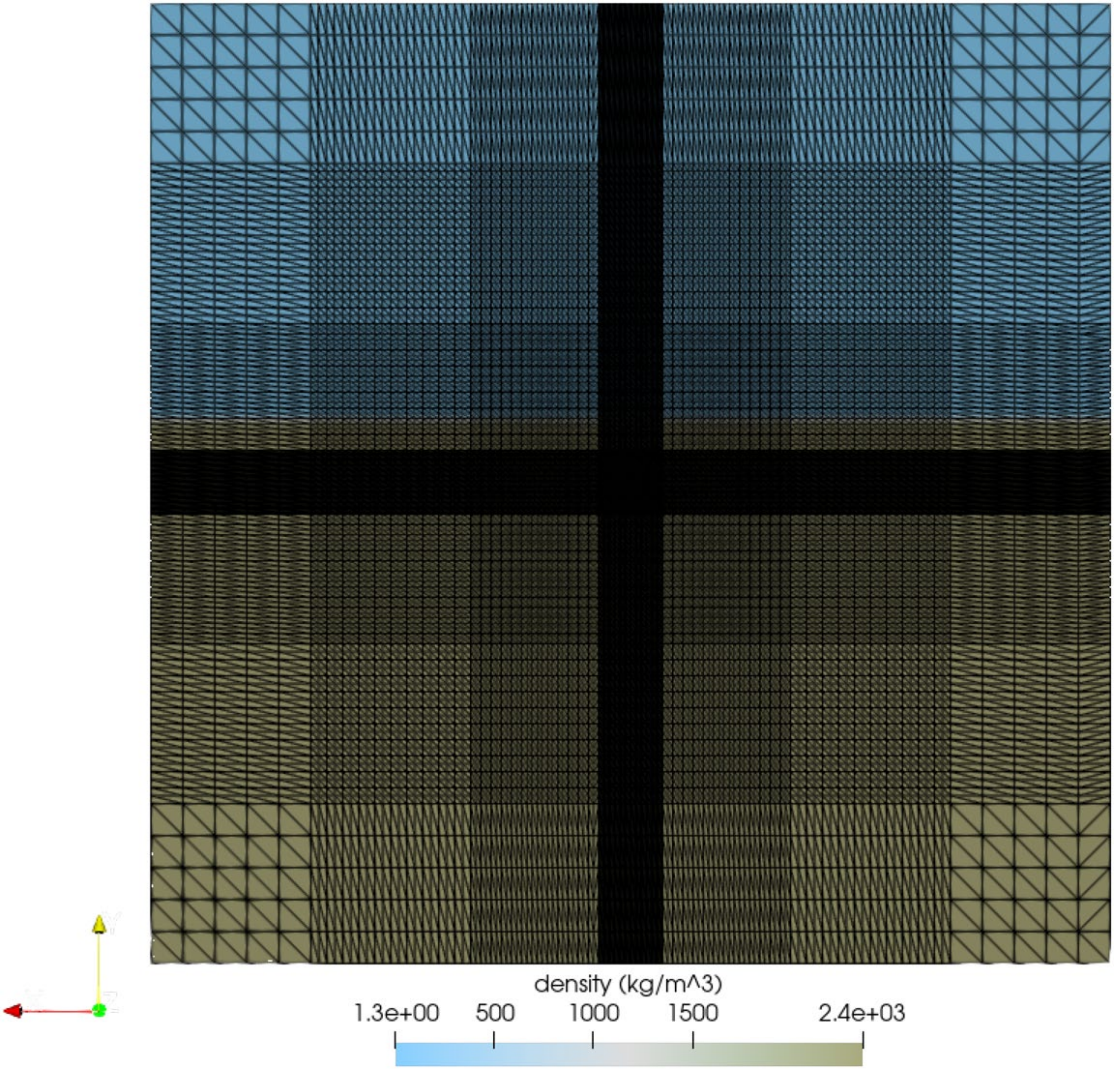
A 2D slice of the 3D FLAG Sedan simulation showing the computational mesh. The mesh was more resolved, with smaller zone sizes, around the source. Farther away, the zones become larger to avoid unnecessarily high computational cost. 2D image from 3D simulation created with ParaView 5.10.1 (https://www.paraview.org/) .

Figure 2 shows the initial mesh for the 3D simulations. Because of the 3D geometry, the figure shows a 2D slice through the center of the computational domain, which includes the center of the source. This view of the 3D simulation at time 0 shows the variety of zone sizes used in this computation. The smaller zones containing the source appear as solid black because of the higher resolution. The zone sizes gradually increase farther away from the center of the source, where residual effects from shock waves have a diminished effect and thus do not require the higher resolution required for modeling the source and subsequent crater.

### HOSS setup

**Figure 3:**
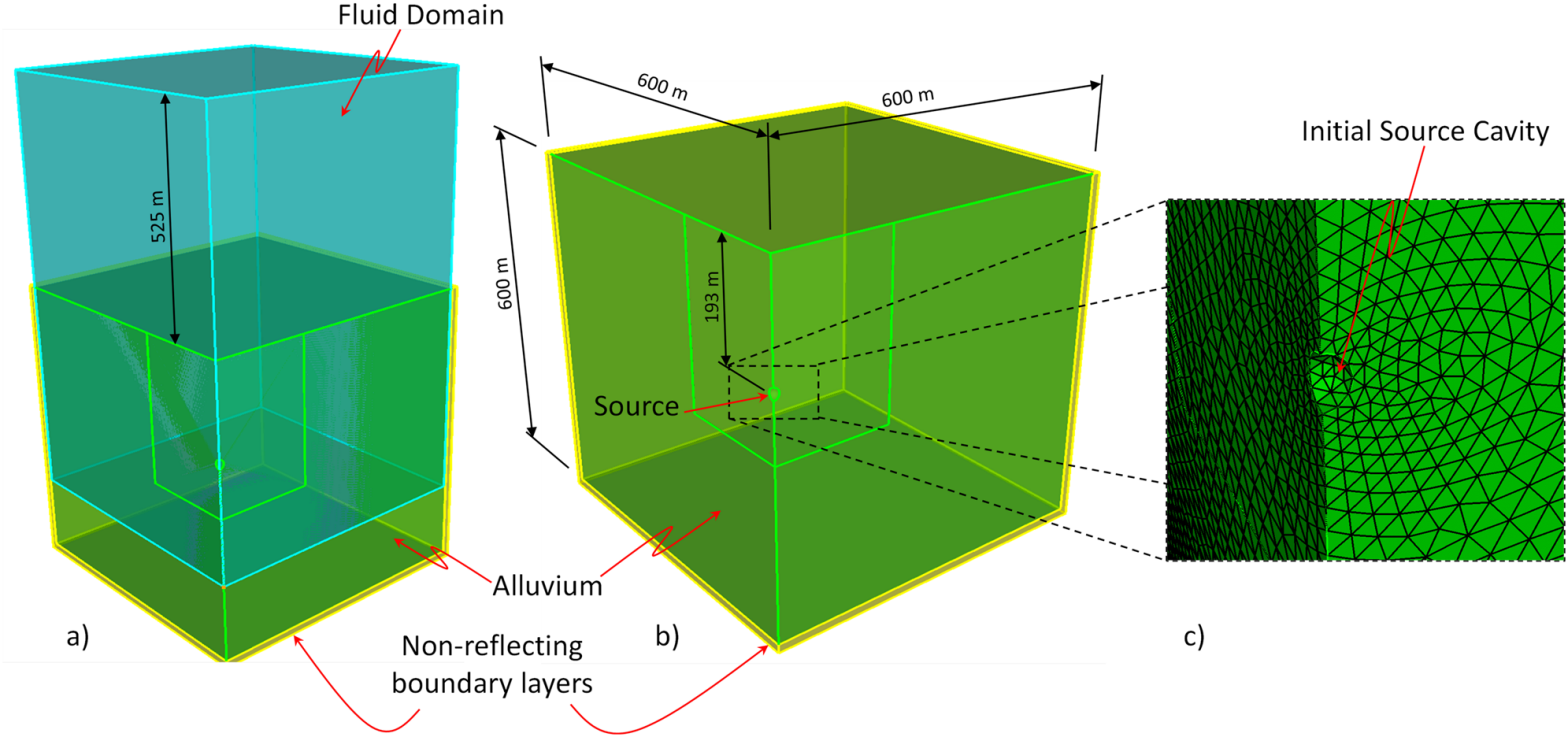
HOSS 3D model setup. (**a**) General view of the model including the solid (ground) and the fluid (air) domains. (**b**) Main dimensions of the ground domain. (**c**) Detailed view of the mesh nearby the initial source cavity area. 3D Figures created using CUBIT 15.8 (https://cubit.sandia.gov/).

The general model setup used in the HOSS simulations is shown in Fig. 3. Because the terrain where the nuclear event occurred was relatively flat, and there are no other relevant asymmetric features, and with the aim of reducing the number of elements and of necessary computational resources needed, a quarter symmetry model was used for this simulation. This information was confirmed by geologic studies of the site conducted at the time, where the alluvium material layer was determined to have a thickness of 396 m^8^. The solid domain was discretized into 666,000 tetrahedral finite elements while the fluid domain was discretized into 20 million hexahedral finite volumes. In this approach, the fluid domain is used to describe the evolution of the source, *i.e.*, vaporized alluvium material, and the surrounding air located above the ground surface. This multi-physics solution allows for the description of the solid response, taking into account the corresponding shear strength of the material and the gas venting when the crater starts to form, via the CFD representation of the source/air regions. The interaction between the solid and the fluid domains is resolved by an extension to 3D of the method introduced by Munjiza *et al.* in^34^. The solid boundary conditions considered a symmetry plane for the surfaces defined by $x=+0.0$ m and $y=+0.0$ m and non-reflecting boundaries surrounding the rest of the model, as shown in Fig. 3.

The whole fluid domain representing the air and the source was modeled using an ideal gas equation of state with a specific heat at constant volume, $C_v$, of 718 J/(kg K) and a specific gas constant, *R*, of 287.2 J/(Kg K). The fluid cells that fall within the vaporization radius were initialized with a density equal to the density of the alluvium, $\rho _{\text {source}}=1710$ kg/m$^3$, and a specific energy corresponding to the yield of the event, $e_{\text {source}}=7.31e7$ J/kg. The boundary conditions for the fluid domain were such that the fluid was allowed to exit the domain of the surfaces defined by $x=+595.0$ m, $y=+595.0$ m, $z=-350.0$ m, and $z=+522.5$ m. This modeling choice means that if the conditions are such that the fluid pressure wave reaches the border of the prescribed domain, then the boundary behaves as an outflow boundary, which minimizes the reflections back into the area of interest. Conversely, no outflow was allowed for the surfaces defined by $x=+0.0$ m and $y=+0.0$ m.

**Figure 4:**
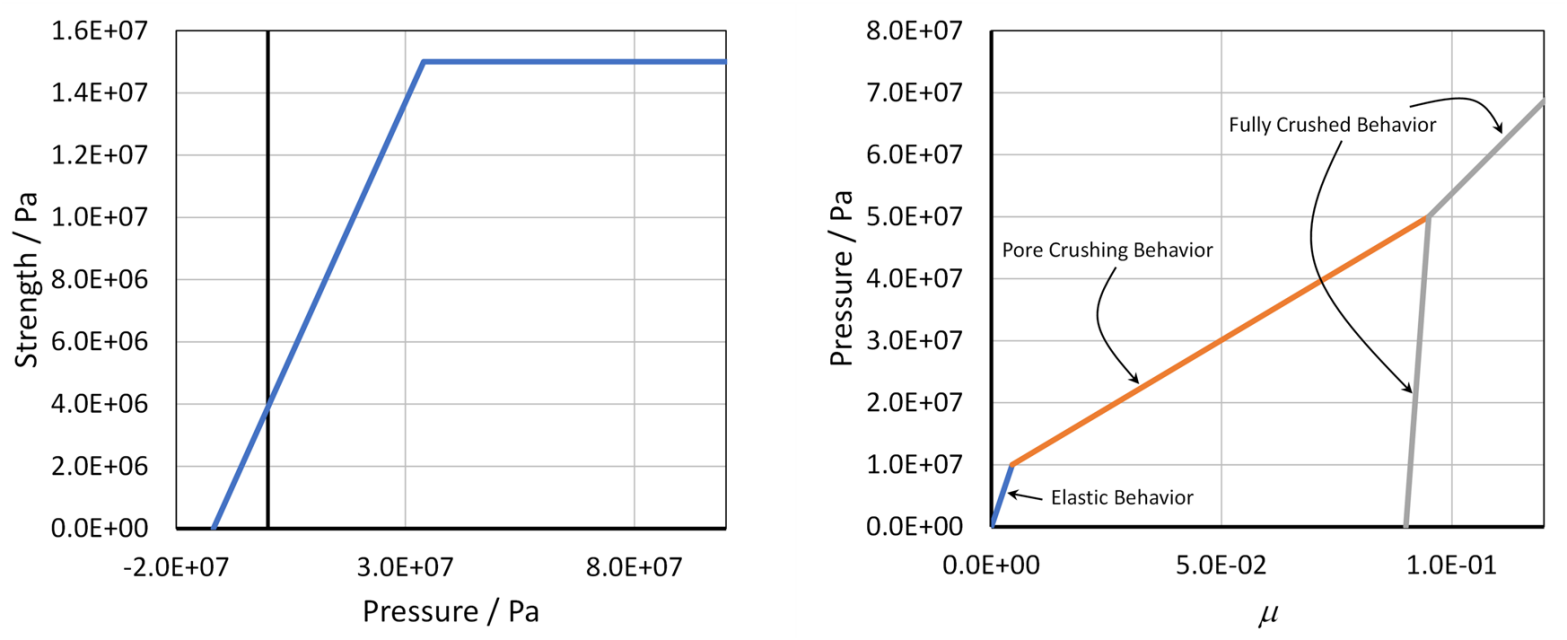
HOSS Material model curves. Left: Yield surface for the 3-invariant strength model. The strength plotted in here corresponds to $\sqrt{3J_2}$, where $J_2$ is the second invariant of the deviatoric stress tensor. Right: EOS curves for alluvium, featuring the elastic, pore crushing, and fully crushed portions of the model. In this graph $\mu =\rho /\rho _0-1$ where $\rho$ and $\rho _0$ are the current and the initial density of the material respectively. Plots created with Microsoft Excel for Microsoft 365 MSO (Version 2503 Build 16.0.18623.20266) 64-bit. (https://www.microsoft.com/en-us/microsoft-365/excel).

The material description used in the HOSS simulations combined an equation of state (EOS) to model the volumetric behavior, including the pore crush process and a 3-invariant strength model that to represent the deviatoric response of the alluvium. The EOS used was derived from a legacy model introduced with the SOC73 code, as detailed in^63^. It allows for elastic volumetric stress until excessive pressure leads to pore space collapse, resulting in permanent volume reduction. Once the pore space is fully compacted, the material can no longer undergo volumetric changes. Consequently, a nonlinear relationship is applied between volumetric strain and volumetric stress^64^. The 3-invariant strength model features a linear elastic deviatoric behavior coupled with a yield surface that depends on pressure, the second and third invariants of the deviatoric stress tensor, and the Lode angle. In both the EOS and strength model, the yield surfaces were user-defined and supplied in tabular format. The density of the alluvium was 1710 kg/m$^3$ and the porosity was 9.6 %, while the bulk and shear moduli were 2.2 GPa and 1.6 GPa, respectively. The tabulated curves used in the EOS and the strength models are shown in Fig. 4. The use of this two-part material description, i.e., EOS featuring pore compaction plus deviatoric strength, is common within the underground explosion modeling community. In particular, the HOSS material model employed in this work was derived from the one used for modeling the MERLIN underground nuclear explosion^65^, which took place on an alluvium geology as well, but on a different part of the Nevada Test Site. However, since the materials for SEDAN and MERLIN are not coming from the same geologic formation, and considering that there was no material characterization data available for the SEDAN geology, some parameter calibration was needed for both the EOS and the strength models for SEDAN. The calibration was performed using available data from geologically similar materials, supplemented by engineering judgment and iterative adjustments to match observed or expected behavior under similar loading conditions. The initial conditions and parameters for the HOSS simulations are listed in Table 2.

**Table 2:** HOSS parameters and initial conditions.

Material property	FLAG initial value
Alluvium (solid)	
Young’s modulus	2.6e9 Pa
Density	1710 kg/m$^3$
Porosity	9.6%
Bulk modulus	2.2e9 Pa
Shear modulus	1.6e9 Pa
Air (gas)	
Pressure	1e5 Pa
Density	1.2922 kg/m$^3$
$\gamma$	1.4
Temperature	273 K
Source (gas)	
Specific heat	718 J/(kg K)
Specific gas constant	287.2 J/(kg K)
Specific energy	7.31e7 J/kg
Density	1710 kg/m$^3$

## 2D axisymmetric results

### FLAG 2D results

We ran a 2D axisymmetric simulation with strength in FLAG to see how a simple isotropic elastic-plastic strength model would compare to experimental data (for preliminary strengthless results. We chose to run in 2D prior to performing a 3D calculation in order to see how accurate a computationally cheaper model would be compared to the actual test. Thus, we were able to have higher resolution in 2D than in 3D. This also allowed us to set up a simpler model to test before moving to the more resource-demanding 3D simulation. An axisymmetric setup was chosen over Cartesian to take advantage of the symmetry in the calculation, as we assume that no notable nonsymmetric features were present in the continuum model.. We expected these results to have smaller errors than the strengthless simulations because of the additional physics included to account for plastic deformation.

**Figure 5:**
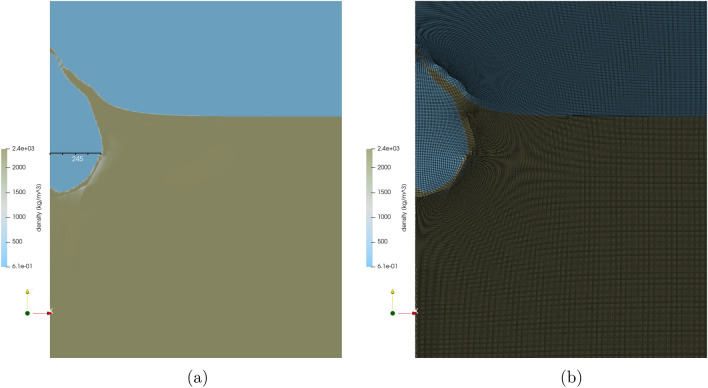
FLAG 2D axisymmetric simulation of Sedan 3 s after detonation, colored by density. At this point in the simulation, the mound is starting to vent, compared to a time of 2.9 s from the actual Sedan test. Figures created using ParaView 5.10.1 (https://www.paraview.org/).

We measured the crater depth at the lowest point in the crater floor. For early simulation times, this point occurs along the axisymmetric boundary. However, in later simulation times, the relaxation of the alluvium coupled with the axisymmetric boundary condition results is a slight uplifting of the alluvium along the boundary, and the deepest point occurs a short distance from the boundary. This rebound behavior can be seen in Fig. 5. We measured crater radius at the widest point along the crater wall, also shown in Fig. 5.

The resulting crater from the simulation after its 5 s run time had a radius of 256 m (relative error of 38%) and a depth of 260 m (relative error of 6% when compared to transient depth). The mound had a height of approximately 73 m above the ground surface at the time of mound rupture. Figure 5 shows the FLAG simulation of Sedan 3 s after detonation, colored by density. The soil vented around 3 s after detonation in the FLAG simulation, compared to about 2.9 s in the actual Sedan test. At the time of venting, the cavity had a radius of about 245 m. This overestimation of the forming crater is expected because the measurement was taken prior to crater modification, the stage during which material falls back and settles into the crater.

**Figure 6:**
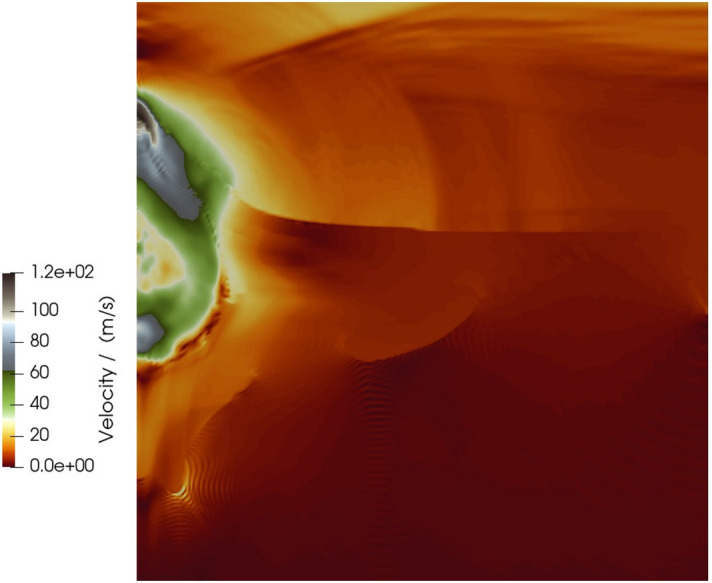
FLAG 2D axisymmetric simulation of Sedan 3.0 s after detonation, colored by velocity magnitude. Figure created using ParaView 5.10.1 (https://www.paraview.org/).

Figure 6 shows the same simulation time colored by speed (velocity magnitude). The mound rupture is evidenced by the moving material, as the alluvium has been uplifted during the explosion. The highest speeds are at the mound rupture site and the bottom of the cavity, along the axisymmetric boundary. At this stage of the simulation, there is little movement in the alluvium away from the detonation point.

**Figure 7:**
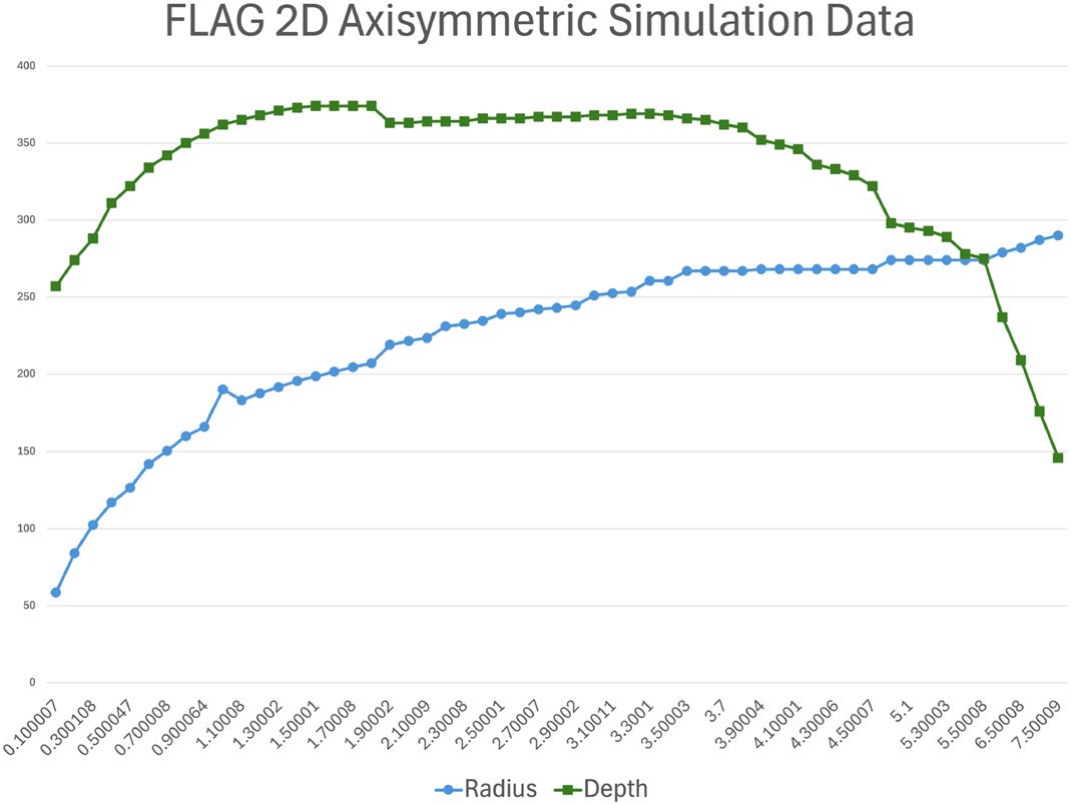
Cavity/crater depth and radius over time for 2D axisymmetric FLAG simulations. The mound ruptured about 2.9 s after detonation in the simulation, at which point the cavity became an open crater. Plot created using Microsoft Excel for Microsoft 365 MSO (Version 2503 Build 16.0.18623.20266) 64-bit. (https://www.microsoft.com/en-us/microsoft-365/excel).

Figure 7 shows crater/cavity depth and radius over time. The mound ruptured at about 2.9 s.

## 3D results

3D calculations were produced for both FLAG and HOSS. While more computationally expensive, a 3D calculation allows material to move more freely across the domain, which produces more physical results than a 2D calculation. Moreover, there are no axisymmetric boundary effects that can exist in a 3D calculation, as there can in a 2D axisymmetric setup. Mesh resolution was varied to reduce computational cost. The mesh was refined to allow for greater accuracy near the source and became more coarse away from the source. Similarly, the HOSS model features a finer solid mesh surrounding the cavity and a progressively coarser mesh away from the source. However, the HOSS fluid mesh which describes the source and subsequent detonation is consistent throughout the model domain.

### FLAG 3D results

**Figure 8:**
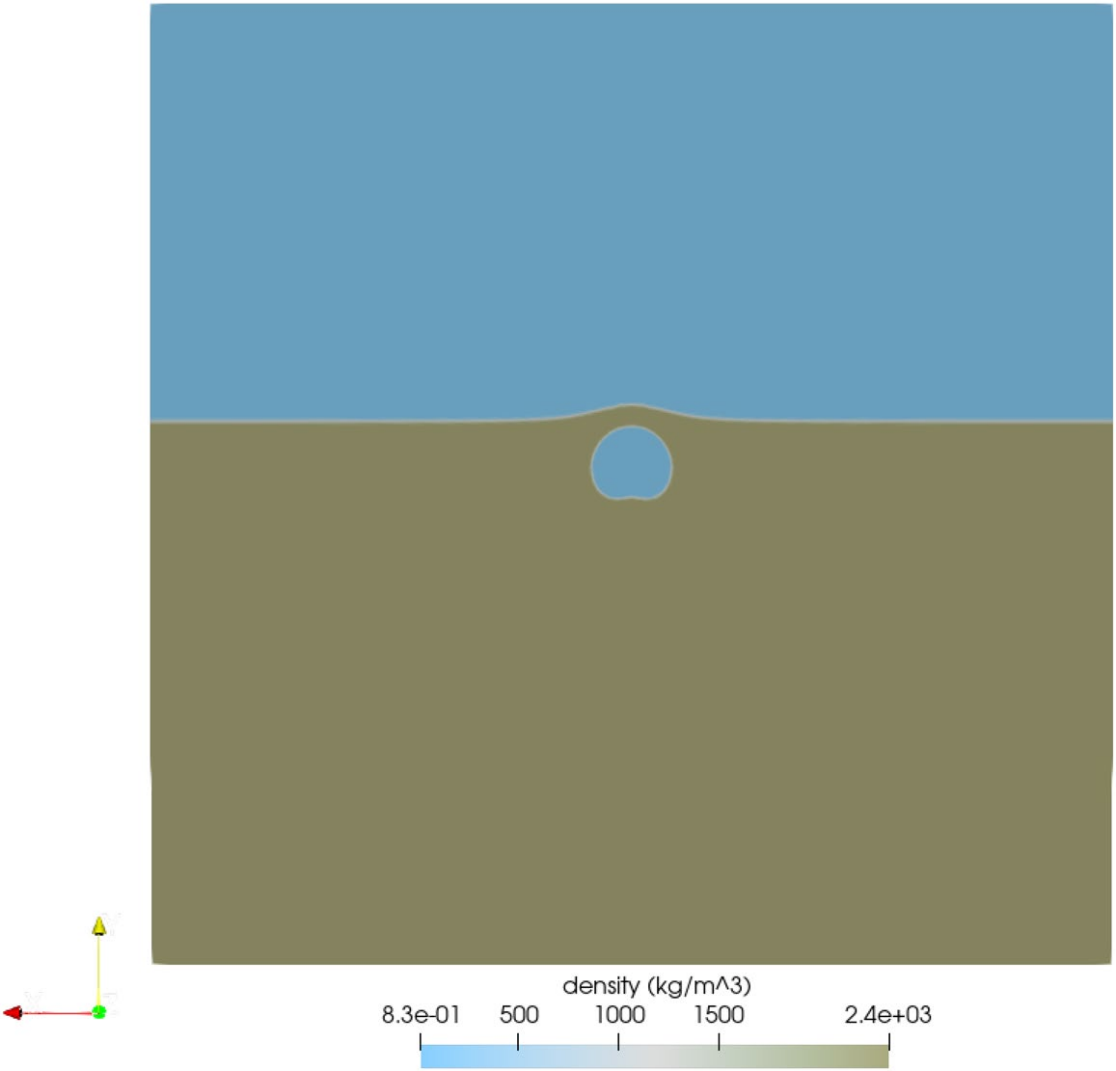
3D FLAG simulation of Sedan, colored by density, 3 s after detonation. 2D image from 3D simulation created with ParaView 5.10.1 (https://www.paraview.org/)..

Figure 8 shows the 3D Sedan simulation in FLAG, colored by density, approximately 3 s after detonation. As the cavity expands, the mound grows until the alluvium yields, resulting in rupture. The 3D geometry allowed for the shock to travel radially outward rather than in the limited directions allowed in 2D. We used the same approach for measuring the cavity and crater as in the 2D case, using a 2D slice through the center of the energy source.

**Figure 9:**
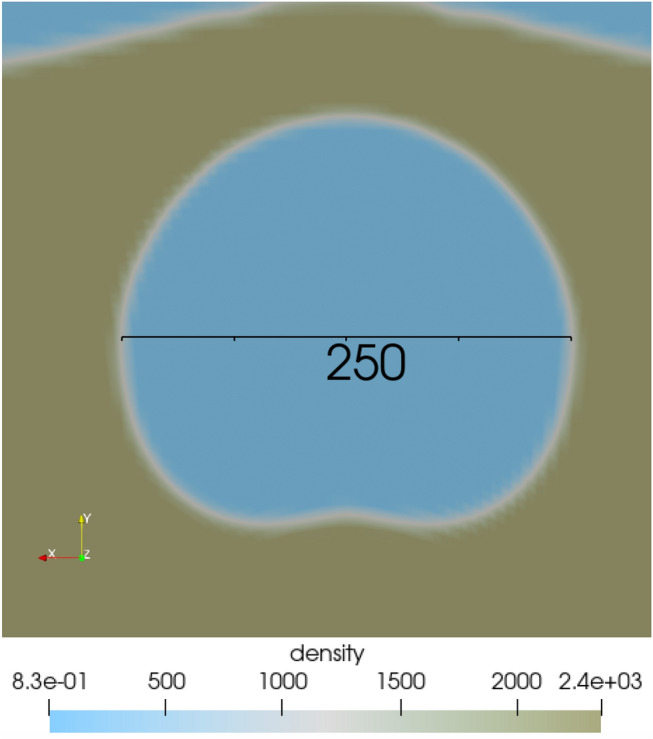
3D FLAG simulation of Sedan 3 s after detonation, showing the cavity. Although the mound has not yet ruptured, the cavity is growing and is about 250 m in diameter at this time. 2D image from 3D simulation created with ParaView 5.10.1 (https://www.paraview.org/).

Figure 9 shows a 2D slice of the 3D FLAG Sedan simulation 3 s after detonation. The cavity grows prior to mound rupture and venting, when the cavity has grown to a diameter of about 250 m. The cavity forms the basis of the crater once the mound ruptures.

**Figure 10:**
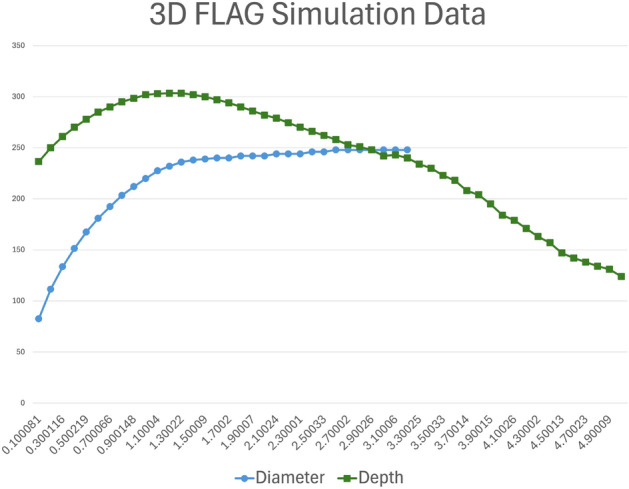
FLAG cavity depth and diameter over time in 3D simulations of the Sedan event. Plot created with Microsoft Excel for Microsoft 365 MSO (Version 2503 Build 16.0.18623.20266) 64-bit. (https://www.microsoft.com/en-us/microsoft-365/excel).

Figure 10 shows cavity dimensions over time. In the 3D simulations, the mound ruptured later than in the 2D simulations, which we attribute to shock attenuation in a third spatial dimension.

We note that the same rebound behavior is present in 3D simulations as in the 2D simulations. In general, this central uplift is expected in very large craters resulting from the slumping of material along the crater wall, and we see this phenomenon in craters on the Martian and lunar surfaces^66^. We also note that we cannot rule out a shock reflection contributing to the small uplift, but we also note that we cannot rule out such behavior as there are clear layers in the alluvium at the Yucca Flat test site^4^.

### HOSS 3D results

**Figure 11:**
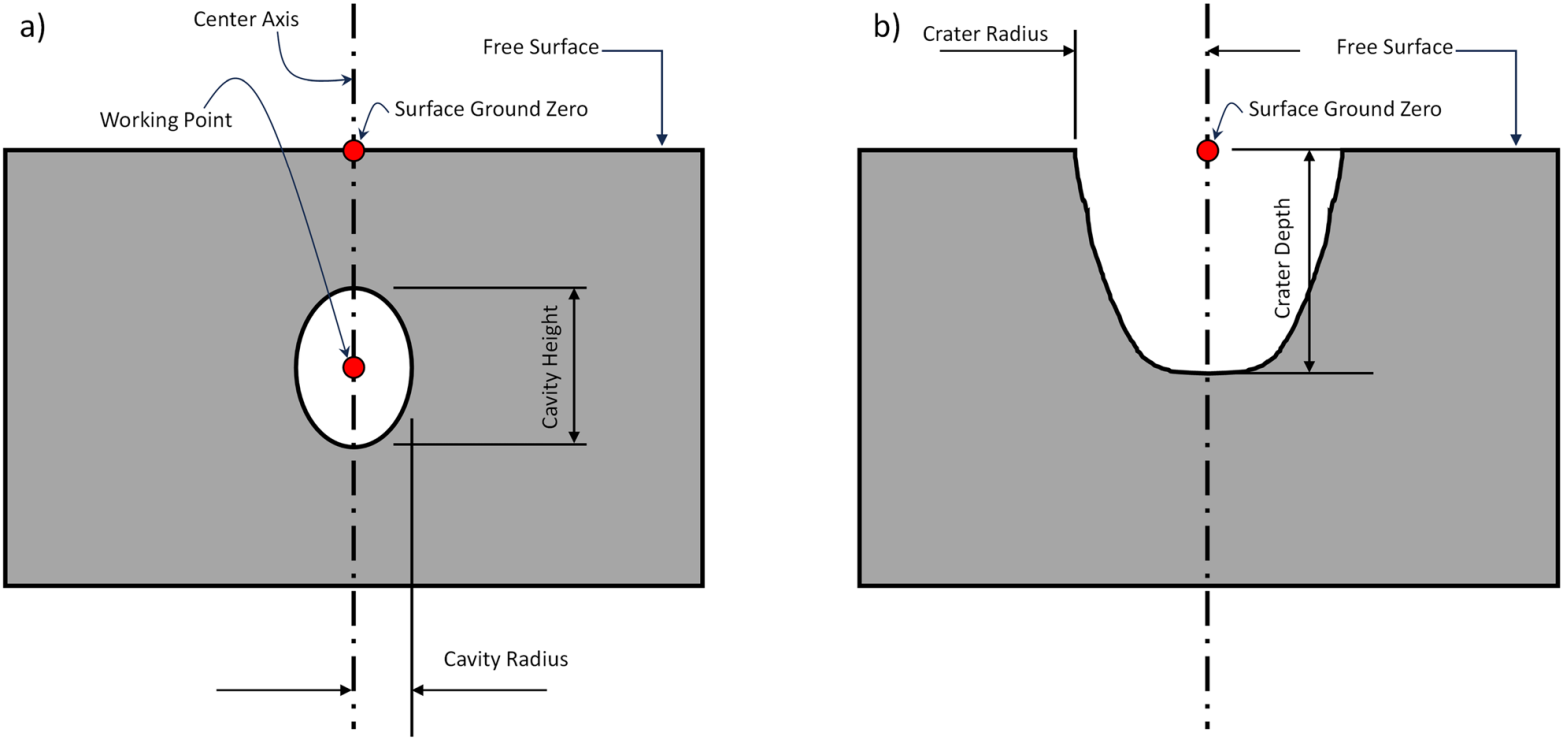
HOSS Metrics for the SEDAN simulation. (**a**) Cavity measurements. (**b**) Crater measurements. Image created using Microsoft PowerPoint for Microsoft 365 MSO (Version 2503 Build 16.0.18623.20266) 64-bit (https://www.microsoft.com/en-us/microsoft-365/powerpoint).

**Figure 12:**
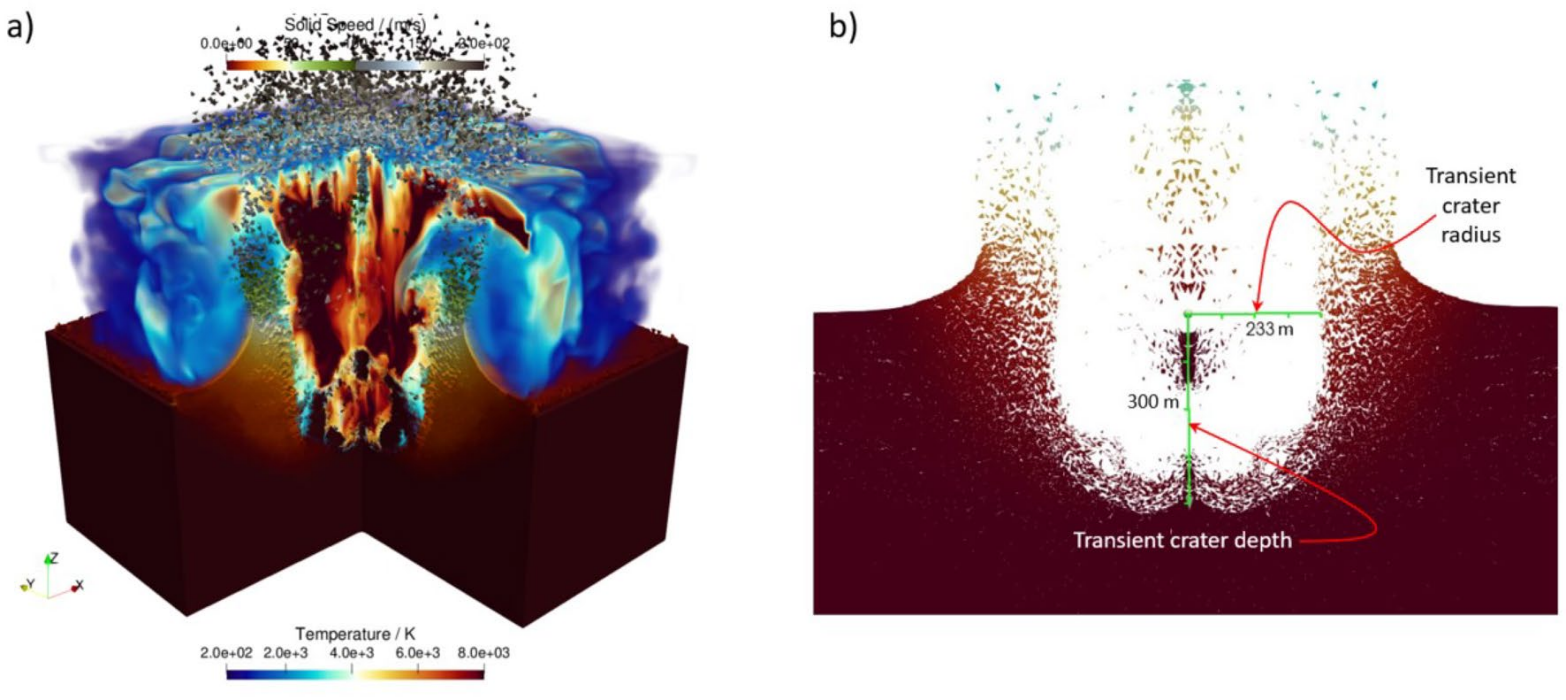
HOSS Final crater dimension. (**a**) General view of the model (mirror images were added for clarity). (**b**) Cross section of the crater with radius and depth transient dimensions. 3D and 2D images created with ParaView 5.10.1 (https://www.paraview.org/).

The HOSS simulations were run using 1210 processors in a high performance computing cluster for 150 hours of wall clock time. The quantities of interest are shown in Fig. 11. The simulation was run for 6.0 seconds and the transient crater dimensions obtained at that point in time are shown in Fig. 12. Two main model features are reported: cavity dimensions and crater dimensions as a function of time. As the cavity grows after the detonation, a mound grows on the free surface which eventually ruptures and creates a crater. The cavity depth is measured from the top to the bottom of the cavity void, while the cavity radius is measured from the working point, as shown in Fig. 11. The crater depth and radius dimensions are measured using a 2D slice of the 3D model taken through the center of the quarter model. The final crater dimensions have a depth of 300m and a radius of 233m. The depth is measured from ground zero to the lowest point at which material is no longer moving. The radius is also measured at ground zero from the center of detonation to the edge of the crater wall. These dimensions do not account for fallback of material after the initial uplift as that occurs at much later times.

**Figure 13:**
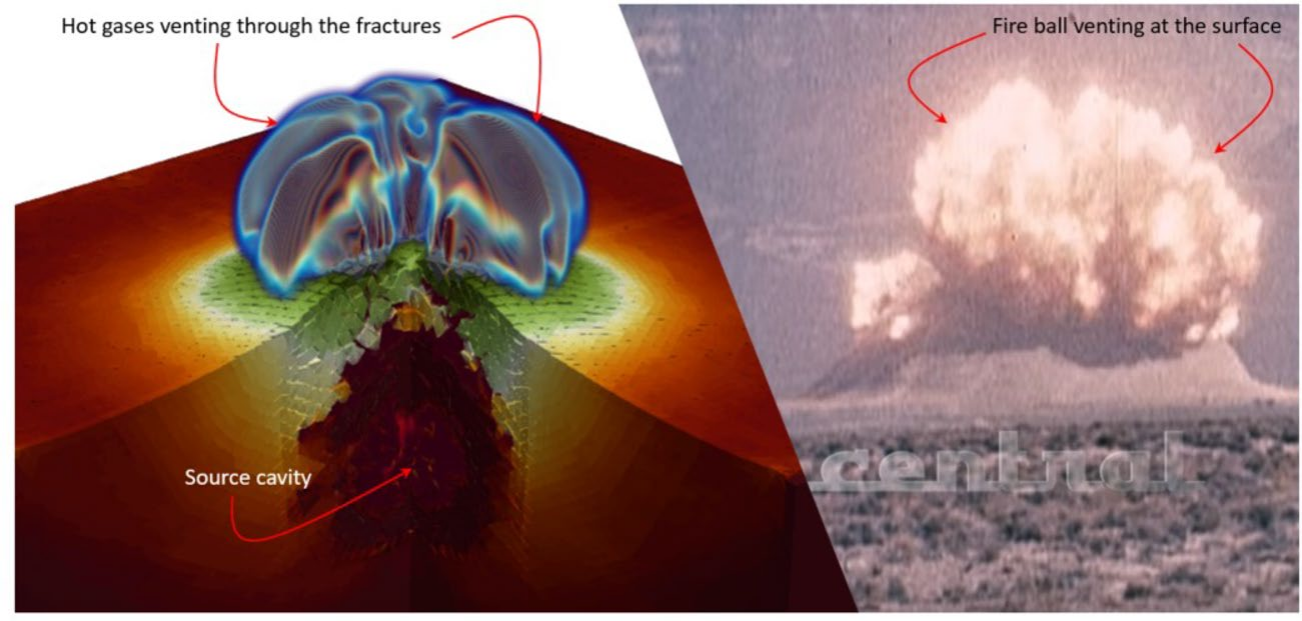
Venting. Left: HOSS results at approximately 2.0 seconds where venting of the hot gases from the source cavity is observed. 3D image created using ParaView 5.10.1 (https://www.paraview.org/). Right: Experimental observation of the venting process recorded at 3.0 seconds. Image from AtomCentral, atomcentral.com, courtesy of the @AtomCentral YouTube Channel. Reprinted with permission.

Shown in Fig. 13 is a comparison of the initial venting in the HOSS simulation and what was recorded from the SEDAN test. In both cases it can be observed that a mound of alluvium rises above the surface which gradually fractures before the hot gases from the source rupture through the fractures. In the HOSS simulation this occurs around 2.0 seconds whereas venting was observed from the SEDAN test at about 3.0 seconds. This differences in venting time can be attributed to the strength of the alluvium material which significantly affects when the mound ruptures.

## Discussion

We note that in this section, we use both “cavity” and “crater” to describe the underground detonation. The cavity refers to the expanding source area prior to mound rupture, while the crater refers to this area once the mound has ruptured. Because we used two different numerical approaches to modeling the formation of the Sedan crater, we do not expect exact matches between the codes. Rather, we expect the FLAG results to match better in early simulation time, when crater/cavity growth is dominated by hydrodynamics. In later simulation time, when material strength becomes the driver of crater size, we expect the HOSS results to be a better match to the actual crater. The crater resulting from the Sedan test had an apparent depth of 98.5 m (after material had settled back into the crater) and a true depth of 245.4 m (prior to crater collapse). The apparent average diameter of the resulting crater was 371.9 m, and a different true diameter was not specified. The true crater volume was estimated to be about 10.6 million m$^3$, equivalent to about 18.2 million tons with an assumed density of 1710 kg/m$^3$ for the alluvium^8^. For this work, we expect successful simulations to result in depths and diameters between the values of the true, pre-modification crater and the settled, apparent crater.

The 2D FLAG simulation had mound rupture 2.9–3.0 s after detonation, consistent with the mound rupture time of the Sedan test. The HOSS 3D simulation observed initial venting at 2.0 s and full mound rupture at 2.75 s. It is around this time that we expect to see the transition from hydrodynamics to strength begin. Prior to mound rupture, both source and material exhibit fluid behavior, which is expected for alluvium under the extreme conditions associated with an underground release of 104 kilotons of energy. After mound rupture, material continues to be ejected from the crater before crater modification occurs, during which time some material falls back into the crater and the crater walls and floor undergo strength-dominated deformation. The 3D FLAG simulation did not result in a ruptured mound by the end of the time at which measurements were taken, about 3.2 s after the explosion. We attribute this behavior to a combination of shock dissipation in an additional spatial dimension, the slightly higher density of the alluvium in the FLAG simulation, and the lack of pore collapse heating from pore compaction, which FLAG and HOSS handle differently. Additionally, the increased computational cost of 3D simulations versus 2D simulations necessitates a coarser mesh with larger zone sizes and a higher number of zones per core during the computation, which has the potential to affect simulation results^12^.

**Figure 14:**
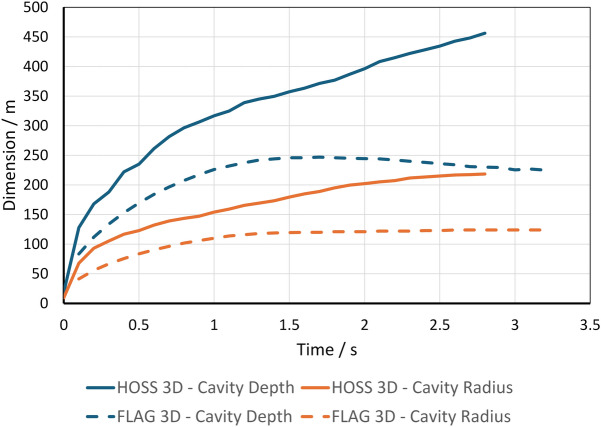
Evolution of the cavity dimensions with time. Image created using Microsoft Excel for Microsoft 365 MSO (Version 2503 Build 16.0.18623.20266) 64-bit. (https://www.microsoft.com/en-us/microsoft-365/excel).

The evolution of cavity growth is graphically shown in Fig. 14 for both the HOSS 3D simulation and the FLAG 3D simulation. In both cases, the depth and radius significantly increase in the initial 0.25 seconds after detonation. Following this initial energy dissipation, the rate of growth of the crater radius gradually decreases until it finally stabilizes. For the HOSS 3D case, the cavity radius nearly reaches its final size at about 2.75 s wherein the radius is 225m. This time also corresponds with when the mound fully ruptures and the cavity is formed into a crater. The crater radius stops growing completely at 4.5 s with a dimension of 233m. The true crater radius is 185 m which yields a percent difference of 25.9% between the true size measured in the SEDAN crater and the HOSS 3D results. The HOSS 3D cavity depth continues to grow more steadily compared to the radius. The cavity depth reaches a maximum dimension of 451 m at 2.75 s before the cavity has fully ruptured and forms a crater. It is worth noting that in previous studies conducted by the authors it was demonstrated that when both codes use strength-less (or near strength-less) material models, a much better agreement in the results is obtained, see^11^.

**Figure 15:**
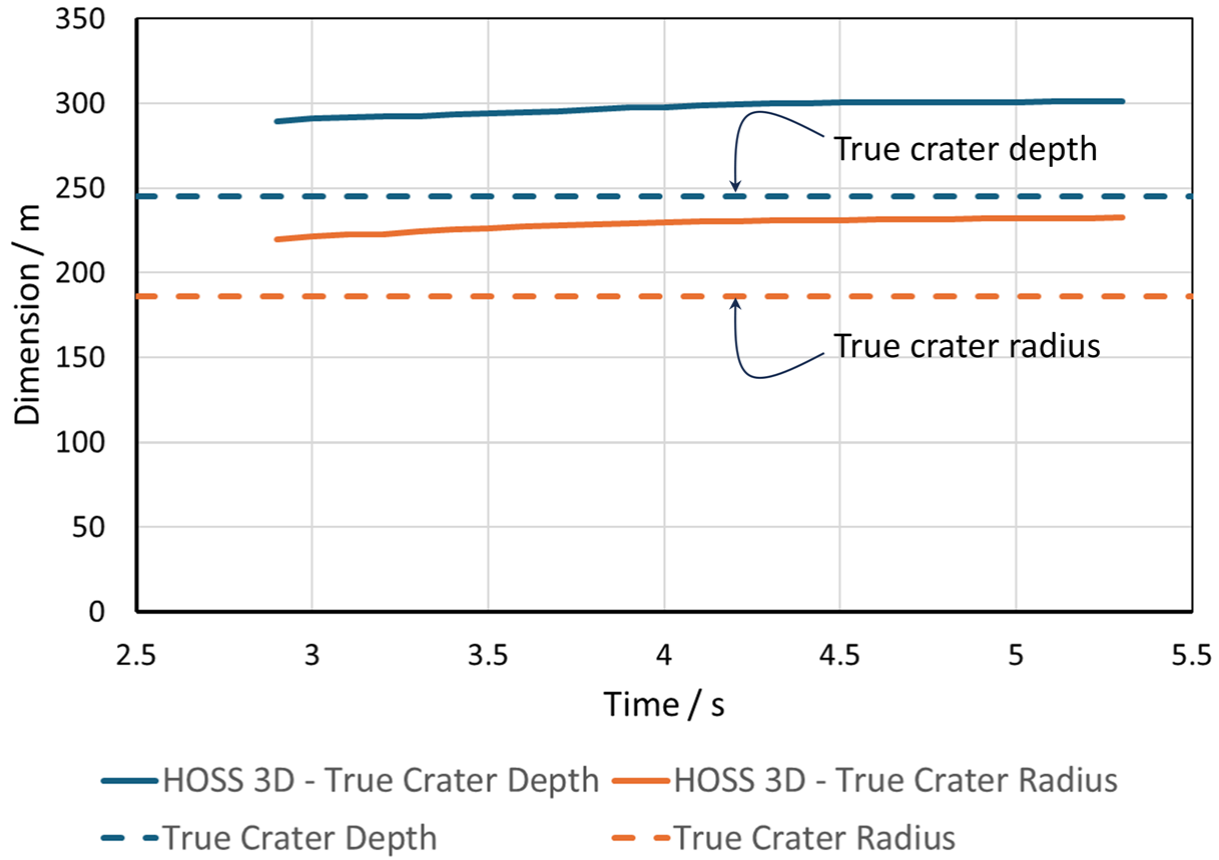
Evolution of the crater dimensions with time. The observed quantities were reported by^8^ and appear as dashed lines. Image created using Microsoft Excel for Microsoft 365 MSO (Version 2503 Build 16.0.18623.20266) 64-bit. (https://www.microsoft.com/en-us/microsoft-365/excel).

The crater evolution in HOSS 3D can be seen in Fig. 15 with the true crater depth and radius shown as reference. The HOSS 3D results overestimates both crater radius and depth by a percent difference of 25.9% and 22.2% respectively. This variance can be attributed to the material strength of the alluvium which also affects the venting time.

**Figure 16:**
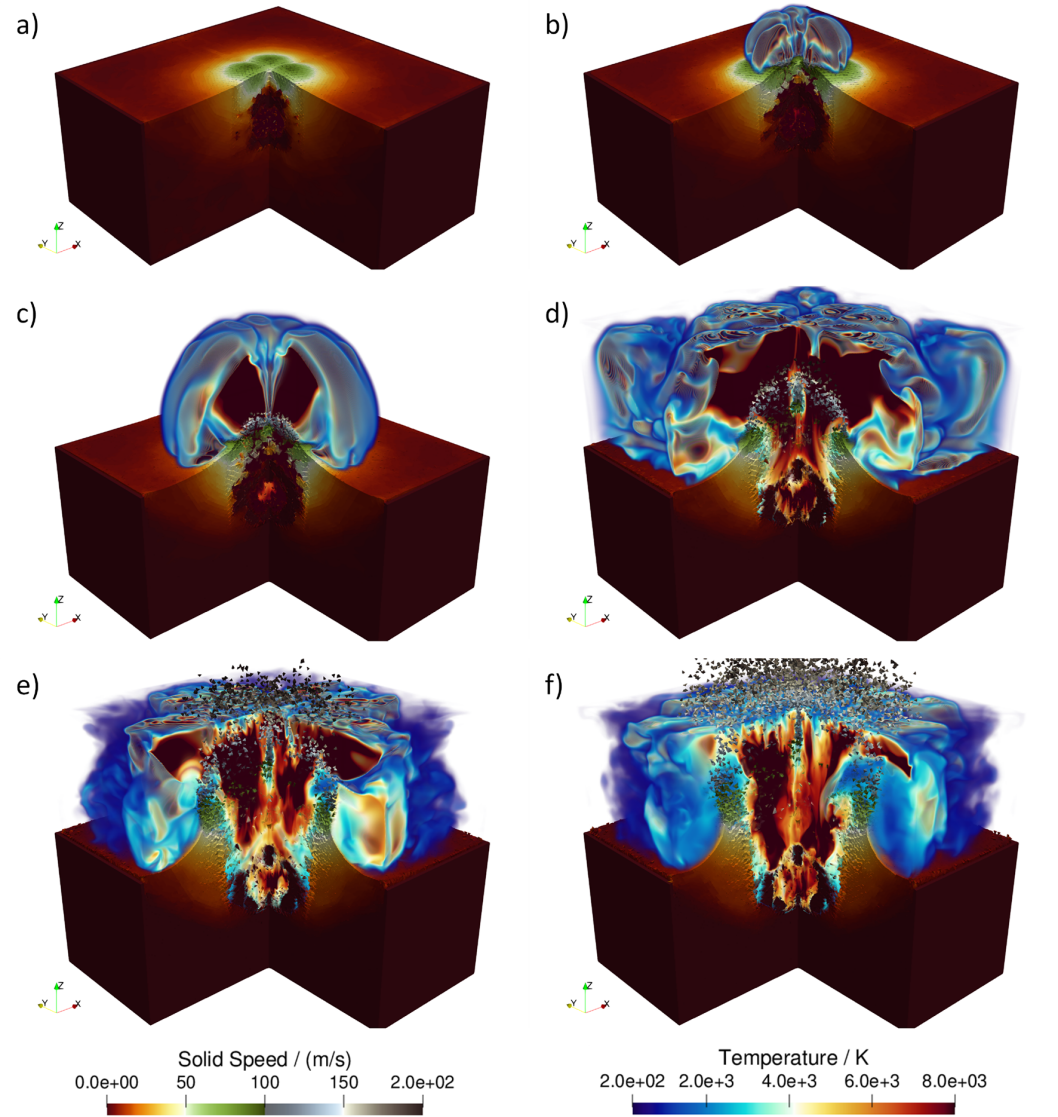
HOSS 3D Simulation. Evolution of the crater formation with time: (**a**) 1 second, (**b**) 2 seconds, (**c**) 3 seconds, (**d**) 4 seconds, (**e**) 5 seconds, and (**f**) 6 seconds. 3D images created with ParaView 5.10.1 (https://www.paraview.org/).

The time evolution of of the HOSS model is shown in Fig. 16. From Fig. 16a it can be seen that at 1.0 s of simulation time a mound is starting to form on the free surface as a consequence of the cavity gases pushing the ground material up. Venting starts at around 2.0 s, as shown in Figure Figure 16b, and the process continues to develop as the simulation progresses, as shown in Fig. 16c–f. In Fig. 16d the shock wave in the fluid reached the top and side boundaries of the computational domain. Because of this, the color map for the fluid temperature appears to be “clipped.” The ejecta formation process (from the material located on top of the cavity) starts at around 3.0 s, as shown in Fig. 16c and continues developing, as shown in Fig. 16d through -f. Eventually, the ejected material is seen flying outside of the fluid domain (Fig. 16e,f); at that point, for those “escaping particles” there is no more interaction being resolved between them and the fluid.

## Conclusions and future work

Our simulations show that a multi-code approach to modeling underground detonations would provide good fidelity for both early simulation time, in which all materials exhibit fluid behavior, and late simulation time, in which solid material properties dominate crater formation. By leveraging the strengths of each approach, we can better understand the entire explosion cratering process.

Our 2D FLAG simulation matched the mound rupture time of the actual test and compared well to the true crater dimensions in early simulation time and would thus be able to provide excellent initial conditions for a later-time 3D HOSS simulation. Further, the 2D FLAG simulation ran to completion in only a few hours, meaning that same-day preliminary results could be obtained for modeling underground detonations.

Our 3D HOSS simulation captured the venting and rupture of the mound and provided a closer match to the final apparent crater size from the Sedan test. HOSS’s ability to accurately model shock physics in geologic materials supports the use of this method for simulation times that align with the strength-dominated stages of crater formation. As shown in this work, the high fidelity provided by HOSS via its explicit fracture and fragmentation formulation and two-way coupling between fluid and solid domains comes at a price in terms of computational time. It is worth noting that since this work was conducted, further developments have been made within HOSS to be able to run 2D axisymmetric models . This new capability will enable researchers to obtain HOSS results with a shorter turn-around time.

Our future work involves validating this multi-code approach to other underground detonations. Additionally, we will be implementing an analytic Tillotson EOS in FLAG to better model heterogenous, layered rocky materials for future simulations. By applying these numerical methods to a novel solution space, we further motivate future work in similar solutions spaces, including cratering on extra-terrestrial bodies, volcanic explosions, and national security applications of these codes.

## Data Availability

The datasets generated and/or analyzed during the current study are not publicly available due to FOIA Exemption 3 and U.S. Export Control Laws but may be made available from the corresponding author through special arrangement.
